# Kinetic modelling of UV_C_ and UV_C_/H_2_O_2_ oxidation of an aqueous mixture of antibiotics in a completely mixed batch photoreactor

**DOI:** 10.1007/s11356-024-34812-7

**Published:** 2024-09-03

**Authors:** Fernando J. Beltrán, Ana M. Chávez, Miguel A. Jiménez-López, Pedro M. Álvarez

**Affiliations:** Departamento de Ingeniería Química y Química Física, Instituto Universitario del Agua, Cambio Climático y Sostenibilidad (IACYS), Universidad de Extremadura, 06006 Badajoz, Spain

**Keywords:** Antibiotics, Hydroxyl radical, Kinetic modelling, Quantum yield, UV_C_/H_2_O_2_ advanced oxidation

## Abstract

The removal kinetics of an aqueous mixture of thirteen antibiotics (i.e., ampicillin, cefuroxime, ciprofloxacin, flumequine, metronidazole, ofloxacin, oxytetracycline, sulfadimethoxine, sulfamethoxazole, sulfamethazine, tetracycline, trimethoprim and tylosin) by batch UV_C_ and UV_C_/H_2_O_2_ processes has been modeled in this work. First, molar absorption coefficients (ε), direct quantum yields (Φ) and the rate constants of the reaction of antibiotics with hydroxyl radical (k_HO•_) (model inputs) were determined for each antibiotic and compared with literature data. The values of these parameters range from 0.3 to 21.8 mM^−1^ cm^−1^ for ε, < 0.01 to 67.8 mmol·E^−1^ for Φ and 3.8 × 10^9^ to 1.7 × 10^10^ M^−1^ s^−1^ for k_HO•_. Second, a regression model was developed to compute the rate constants of the reactions of the antibiotics with singlet oxygen (k^1^_O₂_) from experimental data obtained in batch UV_C_ experiments treating a mixture of the antibiotics. k^1^_O₂_ values in the 1–50 × 10^6^ M^−1^ s^−1^ range were obtained for the antibiotics studied. Finally, a semi-empirical kinetic model comprising a set of ordinary differential equations was solved to simulate the evolution of the residual concentration of antibiotics and hydrogen peroxide (model outputs) in a completely mixed batch photoreactor. Model predictions were reasonably consistent with the experimental data. The kinetic model developed might be combined with computational fluid dynamics to predict process performance and energy consumption in UV_C_ and UV_C_/H_2_O_2_ applications at full scale.

**Supplementary Information:**

The online version contains supplementary material available at 10.1007/s11356-024-34812-7.

## Introduction

The increasing occurrence of different classes of antibiotics in the aquatic environment is considered one of the biggest threats to public health in the XXI century because of the development of antimicrobial resistance (Larsson and Flach 2022). Antibiotics enter the environment mainly through hospital and domestic wastewater treatment plants (WWTPs) due to their incomplete decomposition by the conventional treatment methods currently applied in these facilities (Pandey et al. 2023). Moreover, the development of antibiotic resistant bacteria (ARB) and antibiotic resistant genes (ARG) is favored in WWTPs, where the bacterial population (e.g., activated sludge), being exposed to relatively low concentrations of antibiotics for a long time, finds a suitable environment for horizontal gene transfer (Zieliński et al. 2022). Therefore, the removal of antibiotics from water is becoming an unavoidable task in WWTPs. Among the various treatment methods proposed in the literature, advanced oxidation processes (AOPs), in which reactive oxygen species (ROS) are generated, are considered as a suitable alternative to achieve complete removal of antibiotics and tackle the problem of antimicrobial resistance (Li et al. 2021; Kalli et al. 2023). Particularly, the combination of UV_C_ radiation and hydrogen peroxide (UV_C_/H_2_O_2_) as well as other UV_C_-driven AOPs have been demonstrated effective for removing antibiotics and combating antimicrobial resistance (Wols et al. 2013; Ghosh et al. 2022; Zhang et al. 2022). In the UV_C_/H_2_O_2_ AOP various pathways can be considered for the degradation of aqueous antibiotics. First, direct photolysis, in which the contaminant molecule is broken down as a result of UV_C_ light absorption (da Luz et al. 2022). Also, hydroxyl radical (HO^•^) is directly generated from homolytic cleavage of aqueous H_2_O_2_ under UV_C_ (Li et al. 2017). Owing to its high oxidizing power (E° = 2.8 V/SHE), HO^•^ is capable of degrading most of aqueous micropollutants, including antibiotics (Wojnárovits et al. 2018). Besides, some antibiotics exhibit photosensitizing properties (e.g., fluoroquinolones) giving rise to the release of ROS, such as singlet oxygen (^1^O_2_), which accelerate the photodegradation of pollutants through an indirect photolysis process (Albini and Monti 2003).

Kinetic modelling is a powerful tool to gain insights into the reaction mechanism and improve the performance of chemical processes (e.g., optimization of dose of reagents or reaction time) (Huang and Zhang 2022). Several models have been developed so far aimed at predicting removal rates of aqueous micropollutants in batch UV_C_/H_2_O_2_ photoreactors (Glaze et al. 1995; Crittenden et al. 1999; Rosenfeldt and Linden 2007; Song et al. 2008; Alpert et al. 2010; Audenaert et al. 2011; Wols et al. 2014; Huang et al. 2022; Dang et al. 2024). Typically, a set of ordinary differential equations (ODEs) is proposed for the target contaminants, H_2_O_2_ and photogenerated ROS. For the latter, either the pseudo-steady state (e.g., Glaze et al. 1995) or the unsteady state approach (e.g., Crittenden et al. 1999) has been usually applied. To effectively predict process performance, in addition to chemical and photochemical reactions of the target pollutants, the model needs to consider the multiple effects of natural organic matter (NOM) and inorganic anions present in the aqueous matrix as well as those of transformation products (TPs). All these species might scavenge ROS and/or be competitive light absorbers showing a UV_C_ shielding effect (Cheng et al. 2022). Given the complex nature of water matrices and the large number of TPs in AOPs, it is not feasible to assess the effect of each individual species but probe inorganic compounds (e.g., carbonate and nitrate) and surrogate parameters for NOM (e.g., dissolved organic carbon, DOC and decadic UV absorption coefficient at 310 nm, UVA_310_) have been commonly used for modelling purposes (Song et al. 2008; Audenaert et al. 2011; Wols et al. 2014). Rosenfeldt and Linden (2007) integrated the R_OH,UV_ concept in a UV/H_2_O_2_ model to predict the HO^•^ scavenging nature of background water matrix. R_OH,UV_ was defined as the HO^•^ exposure per UV fluence, being experimentally assessed for a given water matrix and initial H_2_O_2_ concentration using *p*-chlorobenzoic acid as a probe compound. More recently, Huang et al (2022) developed a semi-empirical model that contains a term to account for the overall HO^•^ scavenging capacity of a tested water matrix. This term was quantified experimentally in a mini-fluidic photoreactor using methylene blue as a probe and isopropyl alcohol or *p*-chlorobenzoic acid as a surrogate matrix component. However, the methods outlined above do not succeed in accurately taking account of the ROS scavenging and absorption characteristics of a changing water matrix as the multiple reactions in the UV/H_2_O_2_ AOP progress. Another critical aspect of kinetic models of homogeneous UV_C_ photoreactors in reliably predicting experimental results under a wide range of operating conditions is the knowledge of the light absorption coefficient (ε), the direct quantum yield (Φ) and the rate constants of the reaction of the target compounds with photogenerated ROS (e.g., k_HO•_ and k^1^_O₂_). Table S1, in the supplementary information, shows ε, Φ, k_HO•_ and k^1^_O₂_ values as reported in the literature for the thirteen antibiotics used in this work. As it can be seen, some data are missed (i.e., not reported) while discrepancies are observed in others likely due to the different conditions used in the laboratory (e.g., temperature, pH, UV lamp and photoreactor) and/or the methodology followed for their determination (e.g., direct method vs competitive kinetics) (Wojnárovits et al. 2018).

The main purpose of this work is the development of a simple and reliable semi-empirical kinetic model able to satisfactorily predict the pollutant concentration–time profiles during the course of batch UV_C_ and UV_C_/H_2_O_2_ runs in a completely mixed photoreactor treating a complex aqueous mixture (thirteen antibiotics). First, ε, Φ and k_HO•_ have been systematically determined for the thirteen antibiotics and compared with literature data. Second, the actual concentrations of some ROS have been estimated from experimental data of the removal rate of an internal reference compound. Also, the UV shielding effect of TPs has been quantified by measuring the actual absorbance of the aqueous solution. Therefore, neither photochemical information about TPs and DOC nor additional experiments to determine the overall ROS scavenging capacity of the aqueous solution are required as model inputs. Finally, a set of ODEs has been solved to simulate the evolution of the residual concentration of antibiotics and hydrogen peroxide in a completely mixed batch photoreactor. The kinetic model developed here for a mixture of antibiotics could also be extended to a wide range of aqueous micropollutants and different water matrices. Also, it might be smoothly combined with computational fluid dynamics (CFD) models to predict process performance and energy consumption in UV_C_ and UV_C_/H_2_O_2_ applications (Alpert et al. 2010; Dang et al. 2024).

## Materials and methods

### Chemicals

Ampicillin sodium salt (PanReac, CAS 69–52-3, AMP), cefuroxime sodium salt (Merck, CAS 56238–63-2, CFX), ciprofloxacin (ACROS Organics, CAS 85721–33-1, CIP), flumequine (Merck, CAS 42835–25-6, FLU) metronidazole (TCI, CAS 443–48-1, MTZ), ofloxacin (Merck, CAS 82419–36-1, OFX), oxytetracycline (Alfa Aesar, CAS 79–57-2, OXT), phenol (Merck, CAS 108–95-2, Ph), sodium nitrite (PanReac, 7632–00-0), sulfamethoxazole (Merck, CAS 423–46-6, SMX), sulfamethazine sodium salt (TCI, CAS 1981–58-4, SMZ), sulfadimethoxine (Merck, CAS 122–11-2, SDX), tetracycline (Meck, CAS 60–54-8,TTC), trimethoprim (Merck, CAS 738–70-5, TMP), tylosin tartrate salt (Alfa Aesar, CAS 1405–54-5, TYL), hydrogen peroxide (PanReac, CAS 7722–84-1), *t*-butanol (PanReac AppliChem, CAS 75–65-0) sodium azide (Merck, 26628–22-8), ortho-phosphoric acid (PanReac AppliChem, CAS 7664–38-2), sodium hydroxide (PanReac, 1310–73-2), acetonitrile (PanReac AppliChem, CAS 75–05-8) were used in this study. All solvents and reagents were employed as received without further purification. Ultrapure water was obtained in a Milli-Q Integral system with a resistivity up to 18.2 MΩ·cm.

### Photochemical experiments

The photochemical experiments were performed in an 800 mL cylindrical glass reactor equipped with a 15W low-pressure mercury vapor UV_C_ lamp (Heraeus, model TNN 15–32) emitting mainly at 254 nm. The lamp was placed in the middle of the reactor inside a quartz sleeve provided with a cooling jacket (Rivas et al. 2011). The incident radiation (I_0_) and the effective light path length of reactor (L) were determined using hydrogen peroxide as actinometer following standard procedures described elsewhere (Rabani et al. 2021). Values of 6.9 × 10^–6^ E·L^−1^·s^−1^ and 2.46 cm were found for I_0_ and L, respectively. The apparent and direct quantum yields of the antibiotics at 254 nm were determined by photolysis experiments under UV_C_ radiation in the absence and presence of sodium azide (0.01 M), respectively. Also, photodegradation of antibiotics in the presence of *t*-butanol (0.01 M) was investigated. The rate constant of the reaction of each antibiotic with HO^•^ (k_HO•,i_) was obtained from UV_C_/H_2_O_2_ (0.1 M) experiments, both in the absence and presence of phenol as a reference compound (Wojnárovits et al. 2018). Finally, an aqueous solution containing the thirteen pharmaceuticals listed in Table S1 was prepared in 50 mM phosphate-buffered ultrapure water with a concentration of 10 μM of each antibiotic and 0.05 M of H_2_O_2_ (if needed). Photochemical experiments were carried out with this multicomponent solution, both, in the absence and presence of H_2_O_2_. All photochemical experiments were performed at pH 7 (0.05 M phosphate-buffered aqueous solution) and 20 ± 2 ºC at least in duplicate.

### Analytical methods

The absorption spectra of antibiotics were recorded from 200 to 450 nm using a Shimadzu UV-1800 apparatus and a quartz cuvette (1 or 5 cm path length). The molar absorption coefficient was determined at 254 nm after applying the Beer-Lambert law to spectrometric measurements of stock solutions of the antibiotics at pH 7. The concentrations of antibiotics in water were analyzed using a Shimadzu Prominence UFLC provided with a degassing unit, high pressure pump (LC-20AD), automatic injector, oven and diode array detector. The analysis was performed using a gradient method with a mixture of acidified ultrapure water (H_3_PO_4_ 0.1% v/v) and acetonitrile at 0.6 ml·min^−1^ (Chávez et al. 2023). The concentration of hydrogen peroxide was evaluated spectrophotometrically following the formation of the yellowish pertitanic acid at 405 nm (O’Sullivan and Tyree 2007).

### Mathematical model

The kinetic model comprises a set of ODEs for mass balances of target compounds (i.e., antibiotics) and hydrogen peroxide in a completely mixed batch photoreactor:

1 $$-\frac{{\text{dC}}_{\text{i}}}{\text{dt}}={\text{r}}_{\text{i}}$$

where C_i_ is the concentration of the species *i* (i.e., antibiotics and hydrogen peroxide) and r_i_ its reaction rate, for which three possible contributions have been considered: direct photolysis (r_UVi_) and reactions with photogenerated singlet oxygen (^1^O_2_) and hydroxyl radical (HO^•^):

2 $${\mathrm{r}}_{\mathrm{i}}={\mathrm{r}}_{\mathrm{UVi}}+{\mathrm{r}}_{{1}_{{\mathrm{O}}_{{2}^{\mathrm{i}}}}}+{\mathrm{r}}_{\mathrm{HO}\bullet \mathrm{i}}={\Phi }_{\mathrm{i}}{\mathrm{e}}_{\mathrm{i}}^{\mathrm{a}}+{\mathrm{k}}_{{1}_{{{0}_{2}}^{,\mathrm{i}}}}{\mathrm{C}}_{{1}_{{0}_{2}}}{\mathrm{C}}_{\mathrm{i}}+{\mathrm{k}}_{\mathrm{HO}\bullet ,\mathrm{i}}{\mathrm{C}}_{{\mathrm{HO}}^{\bullet }}{\mathrm{C}}_{\mathrm{i}}$$

where Φ_i_ is the direct quantum yield of the compound *i* at the given wavelength and e^a^_i_ represents the volumetric rate of photon absorption. k^1^_O₂,i_ and k_HO•,i_ are the rate constants of the reactions between the antibiotic *i* and singlet oxygen and hydroxyl radical, respectively, and C^1^_O₂_ and C_HO•_ the actual concentration of these ROS.

The volumetric rate of photon absorption, e^a^_i_, can be obtained from the integration of the radiation transfer equation (Brucato et al. 2006):

3 $${\text{e}}_{\text{i}}^{\text{a}}=\frac{1}{\text{V}}\underset{0}{\overset{\text{L}}{\int }}{\text{e}}_{\text{il}}^{\text{a}}\text{dV}$$

where e^a^_il_ is the local volumetric rate of photon absorption of the compound *i* and V the irradiated reactor volume. In the absence of dispersion of radiation and considering one-dimensional radial radiation and a mono-wavelength radiation source (i.e., 254 nm), Eq. (3) becomes:

4 $${\mathrm{e}}_{\mathrm{i}}^{\mathrm{a}}=\frac{1}{\mathrm{L}}\underset{0}{\overset{\mathrm{L}}{\int }}{\mathrm{k}}_{\mathrm{i}}{\mathrm{G}}_{\mathrm{w}}\mathrm{exp}(-\sum_{\mathrm{j}}{\mathrm{k}}_{\mathrm{j}}{\mathrm{C}}_{\mathrm{j}}\mathrm{r})\mathrm{dr}$$

with L being the effective path length of radiation, G_W_ the surface incident radiation at the reactor wall and k_i_ or k_j_ the absorption coefficient for any *i* or *j* species present in water, respectively. This coefficient can be expressed as a function of the corresponding molar absorption coefficient (ε_i_) as follows:

5 $${\mathrm{k}}_{\mathrm{i}}=2.303{\varepsilon }_{\mathrm{i}}{\mathrm{C}}_{\mathrm{i}}$$

Integration of Eq. (4) leads to Eq. (6):

6 $${\mathrm{e}}_{\mathrm{i}}^{\mathrm{a}}={\mathrm{I}}_{0}{\mathrm{F}}_{\mathrm{i}}\left[1-\mathrm{exp}(-2.303\;\mathrm{ L}\sum_{\mathrm{j}}{\varepsilon }_{\mathrm{j}}{\mathrm{C}}_{\mathrm{j}})\right]$$

where I_0_ is the volumetric incident radiation, F_i_ is the fraction of incident radiation absorbed by the species *i* and Σε_j_C_j_ the absorbance of the aqueous solution measured with a 1 cm quartz cell:

7 $${\text{I}}_{0}=\frac{{\text{G}}_{\text{w}}}{\text{L}}$$

8 $${\mathrm{F}}_{\mathrm{i}}=\frac{{\upvarepsilon }_{\mathrm{i}}{\mathrm{C}}_{\mathrm{i}}}{\sum_{\mathrm{j}}{\varepsilon }_{\mathrm{j}}{\mathrm{C}}_{\mathrm{j}}}$$

9 $$\sum_{\mathrm{j}}{\varepsilon }_{\mathrm{j}}{\mathrm{C}}_{\mathrm{j}}=\mathrm{A}$$

Then, the final mass balance equation to be solved for each species *i* is:

10 $$-\frac{{\mathrm{dC}}_{\mathrm{i}}}{\mathrm{dt}}={\mathrm{r}}_{\mathrm{UVi}}+{\mathrm{r}}_{{1}_{{\mathrm{O}}_{{2}^{\mathrm{i}}}}}+{\mathrm{r}}_{{\mathrm{HO}}^{\bullet }\mathrm{i}}=\frac{{\mathrm{I}}_{0}{\Phi }_{\mathrm{i}}{\varepsilon }_{\mathrm{i}}{\mathrm{C}}_{\mathrm{i}}}{\mathrm{A}}\left[1-\mathrm{exp}\left(-2.303\mathrm{LA}\right)\right]+{\mathrm{k}}_{{1}_{{{0}_{2}}^{,\mathrm{i}}}}{\mathrm{C}}_{{1}_{{0}_{2}}}{\mathrm{C}}_{\mathrm{i}}+{\mathrm{k}}_{{\mathrm{HO}}^{\bullet },\mathrm{i}}{\mathrm{C}}_{{\mathrm{HO}}^{\bullet }}{\mathrm{C}}_{\mathrm{i}}$$

with initial conditions:

11 $$\begin{array}{cc}\mathrm{t}=0& \begin{array}{cc}{\mathrm{C}}_{\mathrm{i}}={\mathrm{C}}_{\mathrm{i}0}& \mathrm{A}={\mathrm{A}}_{0}=\sum_{\mathrm{j}}{\varepsilon }_{\mathrm{j}}{\mathrm{C}}_{\mathrm{j}0}\end{array}\end{array}$$

## Results and discussion

### UV absorption spectra

**Fig. 1:**
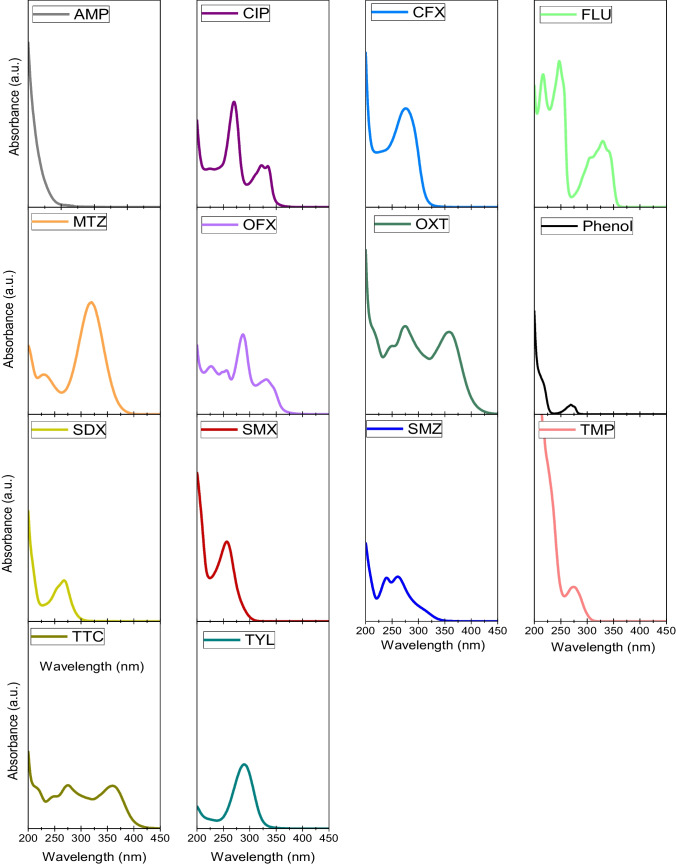
Absorption spectra (200–400 nm wavelength) of aqueous solutions of individual antibiotics and phenol at pH 7

Antibiotics may be degraded in aqueous solution under illumination at different wavelengths. The absorption spectra of the aqueous solution of an antibiotic gives a hint about whether a radiation source is useful for its direct photolysis (Pereira et al. 2007; Lian et al. 2015). Figure 1 gathers the absorption spectra recorded for aqueous solutions of individual antibiotics at pH 7. As seen, all the compounds considered in this work absorb radiation of 254 nm to some extent, though the maximum of absorption is shifted towards higher wavelengths in some cases (e.g., CIP, MTZ or TYL). Table 1 summarizes the molar absorption coefficients as measured at pH 7. If compared with those shown in Table S1 (literature data), good agreement was found for AMP, CIP, MTZ, OFX, OXT, SMX and TYL (deviation < 20%). No reported data were found in the available literature for CFX, FLU, SDX, SMZ, TMP and TTC at pH 7 (see Table S1).

**Table 1:** Molar absorption coefficient and quantum yields at 254 nm of aqueous solutions of some antibiotics

Antibiotic	ε (mM^−1^·cm^−1^)	Φ_app_ (mmol·E^−1^)^a^	Φ_d_ (mmol·E^−1^)^b^
AMP	0.33	69.1	67.8
CFX	13.6	38.3	36.5
CIP	17.9	4.8	1.7
FLU	18.6	0.6	< 0.01
MTZ	1.9	3.7	1.3
OFX	14.7	4.1	1.9
OXT	11.2	4.7	4.5
SDX	21.8	5.8	4.6
SMX	16.6	14.9	10.9
SMZ	16.1	2.3	2.0
TMP	4.6	0.3	< 0.01
TTC	11.0	4.0	3.8
TYL	4.5	1.9	1.5

^a^ Apparent quantum yield; ^b^ Direct quantum yield. Experimental conditions as in Fig. 2

### Apparent and direct quantum yields

Literature reports that some antibiotics (e.g., fluoroquinolones) and other organics such as dyes, insecticides or quinones have photosensitizing properties (Alegría et al. 1999, Wang et al. 2023, Albini and Monti 2003) so that while being photolyzed in aqueous solution they generate ROS (mainly singlet oxygen and hydroxyl radical) through an excited triplet state mechanism. As a result, their photolysis and those of other species in solution are accelerated (i.e., indirect photolysis). For instance, photolysis of the fluoroquinolone OFX yields various ROS according to reactions (12) to (18) (Rodríguez et al. 2015):

12 $$\text{OFX}\stackrel{\text{hv}}{\to }{}^{1}{\text{OFX}}^{*}$$

13 $${}^{1}{\text{OFX}}^{*}\to {}^{3}{\text{OFX}}^{*}$$

14 $${}^{3}{\text{OFX}}^{*}+{\text{O}}_{2}\to \text{OFX}+{}^{1}{\text{O}}_{2}$$

15 $${}^{3}{\text{OFX}}^{*}+{}^{1}{\text{O}}_{2}\to {\text{OFX}}^{\bullet +}+{\text{O}}_{2}^{\bullet -}$$

16 $${2\text{O}}_{2}^{\bullet -}+{2\text{H}}^{+}\to {\text{H}}_{2}{\text{O}}_{2}+{}^{1}{\text{O}}_{2}$$

17 $${\text{H}}_{2}{\text{O}}_{2}\stackrel{\text{hv}}{\to }{2\text{HO}}^{\bullet }$$

18 $${\text{HO}}^{\bullet }+{\text{O}}_{2}^{\bullet -}\to {\text{OH}}^{-}+{}^{1}{\text{O}}_{2}$$

Once ROS are formed, they can oxidize organic compounds in parallel to direct photolysis reactions. If it is assumed that ^1^O_2_ and HO^•^ are the main ROS generated, Eq. (10) can be applied to a self-sensitized process, being Φ_i_ the direct quantum yield. In the presence of ROS quenchers, Eq. (10) can be simplified to Eq. (19):

19 $$-\frac{{\mathrm{dC}}_{\mathrm{i}}}{\mathrm{dt}}={\mathrm{r}}_{\mathrm{UVi}}=\frac{{\mathrm{I}}_{0}{\Phi }_{\mathrm{i}}{\upvarepsilon }_{\mathrm{i}}{\mathrm{C}}_{\mathrm{i}}}{\mathrm{A}}\left[1-\mathrm{exp}(-2.303\mathrm{LA})\right]$$

Once I_0_, L and ε_i_ are known (see experimental section for I_0_ and L and Table 1 for ε_i_), the determination of the direct quantum yield of the antibiotics under study can be accomplished through individual photolysis experiments in the presence of ROS scavengers by applying Eq. (19) in its integrated form:

20 $$Y={C}_{i0}-{C}_{i}-\frac{1}{2.303{L\varepsilon }_{i}}\text{ln}\left[\frac{1-\text{exp}(-2.303{L\varepsilon }_{i}{C}_{i}) }{1-\text{exp}(-2.303{L\varepsilon }_{i}{C}_{i0})}\right]={I}_{0}{\Phi }_{i}t$$

**Fig. 2:**
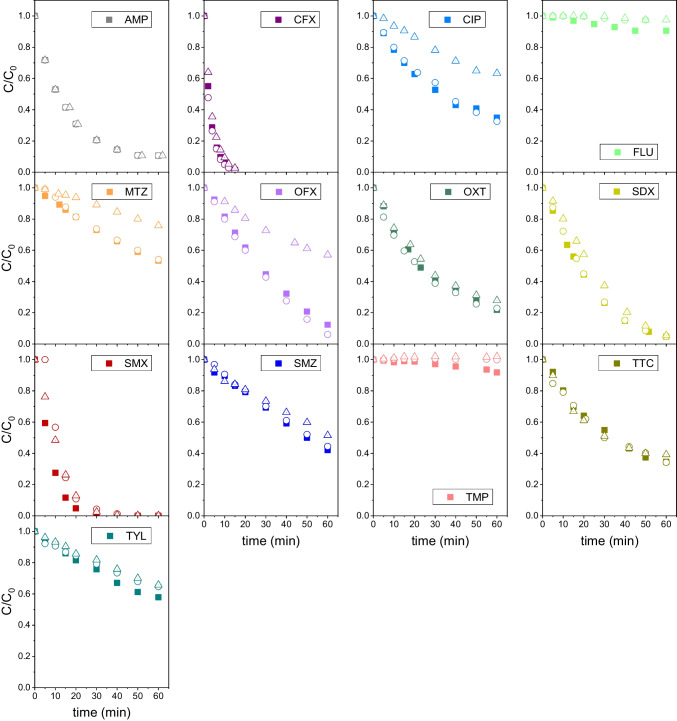
Evolution of dimensionless concentration of antibiotics with time during their individual photolysis under UV_C_ radiation. Symbols: No scavenger used (solid symbols); *t-*butanol 0.01 M (open circles); NaN_3_ 0.01 M (open triangles). Conditions: T = 18–20 ºC, pH 7, C_0_ = 0.1 mM; I_0_ = 6.9 × 10^–6^ E·L^−1^·s^−1^, L = 2.46 cm

It should be pointed out that for integration of Eq. (19), the condition A = ε_i_C_i_ was applied. This means that the fraction of incident radiation absorbed by species other than the antibiotic *i* is considered negligible. Equation (20) can also be used in the absence of ROS scavengers to estimate an apparent quantum yield (i.e., total number of molecules of *i* reacted per mole of absorbed photons), which accounts for the overall removal of the antibiotic by both direct and indirect photolysis pathways. In this work, individual photolysis runs of the thirteen antibiotics under study were carried out in the presence and absence of *t-*butanol (scavenger of HO^•^) or sodium azide (scavenger of both HO^•^ and ^1^O_2_) to account for the contribution of indirect photolysis due to hydroxyl radical and singlet oxygen to the apparent quantum yield. Figure 2 show the evolution of the normalized concentration of antibiotic with the irradiation time during sixty-minute photolysis runs of aqueous solutions of individual antibiotics. It can be seen that regardless of the presence of any ROS scavenger, FLU and TMP were barely degraded with less than 10% removal in 1 h. As both substances can absorb UV_C_ radiation to some extent (see ε values in Table 1), their slow removals in the runs suggest very low direct and apparent quantum yields for them. AMP, CFX, OXT and TTC showed different photoreactivity with removals in the range of about 65% (TTC) to 100% (CFX). However, in all these cases from subtle to no effect of the presence of sodium azide or *t*-butanol was observed, suggesting that no indirect photolysis took place. Likewise, the sulfonamide antibiotics were removed to different extents, from about 60% (SMZ) to 100% (SMX), being the impact of the ROS quenchers minor. On the contrary, sodium azide highly suppressed the removal of CIP, MTZ and OFX while *t*-butanol did not exert any inhibition action, suggesting that singlet oxygen, rather than hydroxyl radical, plays a key role in the indirect photolysis mechanisms of these antibiotics. Finally, sodium azide and *t*-butanol inhibited to a similar extent the photolysis of TYL, indicating that HO^•^ might be involved in the photooxidation of this compound under UV_C_ radiation.

**Fig. 3:**
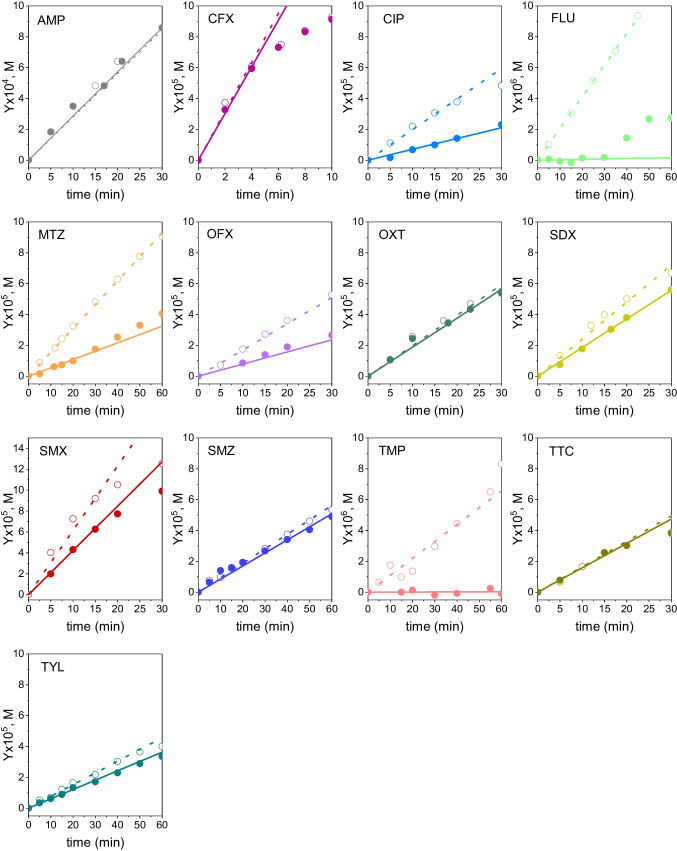
Determination of apparent and direct quantum yield of antibiotics by regression of experimental data to Eq. (20). Symbols: No scavenger used (open symbols); NaN_3_ 0.01 M (solid symbols). Experimental conditions as in Fig. 2

Equation (20) was applied to data of Fig. 2 to obtain the quantum yields, both in the presence of sodium azide (direct quantum yield, Φ_d_) and the absence of ROS scavengers (apparent quantum yield, Φ_app_). As it can be seen in Fig. 3, experimental data follow linear trends though some deviations are observed at long reaction times in some instances (see SDX graph as an example). This is likely due to the effects of reaction intermediates, which are not accounted for in Eq. (20). However, a good linearity is observed within the first minutes of any experiment, allowing the determination of the quantum yield by linear regression. The marked gap between apparent and direct quantum yields listed in Table 1 for CIP, FLU, MTZ, OFX and TMP corroborates the importance of the indirect photolysis to the overall UV_C_ degradation of these compounds. This is especially significant in the cases of fluoroquinolone antibiotics (i.e., CIP, OFX and FLU), confirming their photosensitizing character (Albini and Monti 2003). Sulfonamides (SDX, SMX and SMZ) and TYL showed apparent quantum yields slightly higher than their corresponding direct quantum yields, suggesting a minor impact of the singlet oxygen and/or hydroxyl radical reactions on the overall photolysis rate. Finally, no indirect photolysis was observed for AMP, CFX and tetracyclines (OXT and TTC) as they resulted in similar apparent and direct quantum yields.

### Hydroxyl radical rate constant of antibiotics

The knowledge of the rate constant of the reaction between any organic compound and the hydroxyl radical is crucial for kinetic modelling of AOPs. The hydroxyl radical rate constant can be determined by different approaches, including laboratory and artificial intelligence methods (Wols and Hofman-Caris 2012; Keivanimehr et al. 2021). The photolysis of H_2_O_2_ under UV_C_ radiation (mainly 254 nm) remains the most widely used as it leads to direct formation of hydroxyl radical, which further reacts with organics constituting the main pathway for their removal. In this work, three different experimental approaches based on the UV_C_/H_2_O_2_ method were used to assess k_HO•,i_ for a selection of antibiotics. First, a numerical method aimed at solving the mass balance equation of the antibiotic in a UV_C_/H_2_O_2_ perfectly mixed batch photoreactor was considered. The Eq. (10) was simplified by neglecting the contribution of singlet oxygen reactions to the overall antibiotic removal:

21 $$-\frac{{\mathrm{dC}}_{\mathrm{i}}}{\mathrm{dt}}=\frac{{\mathrm{I}}_{0}{\Phi }_{\mathrm{i}}{\upvarepsilon }_{\mathrm{i}}{\mathrm{C}}_{\mathrm{i}}}{\mathrm{A}}\left[1-\mathrm{exp}\left(-2.303\mathrm{LA}\right)\right]+{\mathrm{k}}_{\mathrm{HO}\bullet ,\mathrm{i}}{\mathrm{C}}_{\mathrm{HO}\bullet }{\mathrm{Ci}}$$

In a UV_C_/H_2_O_2_ oxidation run of an individual antibiotic *i*, the concentration of hydroxyl radical can be calculated after applying the pseudo-steady state approach to this ROS (Beltrán 2004):

22 $${\text{C}}_{\text{HO}\bullet }=\frac{{2\text{I}}_{0}{\Phi }_{{\text{H}}_{2}{\text{O}}_{2,\text{T}}}{\upvarepsilon }_{{\text{H}}_{2}{\text{O}}_{2,\text{T}}}{\text{C}}_{{\text{H}}_{2}{\text{O}}_{2},\text{T}}}{{\text{k}}_{\text{HO}\bullet \text{i}}{\text{C}}_{\text{i}}+{\text{k}}_{\text{HO}\bullet {\text{H}}_{2}{\text{O}}_{2,\text{T}}}{\text{C}}_{{\text{H}}_{2}{\text{O}}_{2,\text{T}}}}\left(\frac{1-\text{exp}(-2.303\text{LA})}{\text{A}}\right)$$

where the subscript H_2_O_2,T_ stands for total hydrogen peroxide (both, molecular and ionic forms) considering the acid–base equilibrium:

23 $${\text{H}}_{2}{\text{O}}_{2}\stackrel{\stackrel{\text{pK}=11.8}{\to }}{\leftarrow }{\text{H}}^{+}+{\text{HO}}_{2}^{-}$$

Thus, Φ _H2O2,T_ and ε_H2O2,T_ are the quantum yield and the molar absorption coefficient of hydrogen peroxide at the working pH. At pH 7, values of Φ _H2O2,T_ = 0.54 mmol·E^−1^ (Rabani et al. 2021) and ε_H2O2,T_ = 19.6 mM^−1^ cm^−1^ (Glaze et al. 1987) are reported. k_HO•,H2O2,T_ is the overall rate constant of the reactions of HO^•^ with H_2_O_2_ and HO_2_^‾^ (Christensen et al. 1982):

24 $${\text{H}}_{2}{\text{O}}_{2}+{\text{HO}}^{\bullet }\stackrel{{\text{k}}_{1}=2.7\times {10}^{7}{\text{M}}^{-1}{\text{s}}^{-1}}{\to }{\text{HO}}_{2}^{\bullet }+{\text{H}}_{2}\text{O}$$

25 $${\text{HO}}_{2}^{-}+{\text{HO}}^{\bullet }\stackrel{{\text{k}}_{2}=7.5\times {10}^{9}{\text{M}}^{-1}{\text{s}}^{-1}}{\to }{\text{HO}}_{2}^{\bullet }+{\text{OH}}^{-}$$

26 $${\text{k}}_{\text{HO}\bullet {\text{H}}_{2}{\text{O}}_{2,\text{T}}}=\frac{{\text{k}}_{1}+{\text{k}}_{2}{10}^{\text{pH}-\text{pKa}}}{1+{10}^{\text{pH}-\text{pKa}}}$$

Substituting the Eq. (22) into the Eq. (21):

27 $$-\frac{{\text{dC}}_{\text{i}}}{\text{dt}}=\left({\Phi }_{\text{i}}{\upvarepsilon }_{\text{i}}+\frac{{2\text{k}}_{\text{HO}\bullet \text{i}}{\Phi }_{{\text{H}}_{2}{\text{O}}_{2,\text{T}}}{\upvarepsilon }_{{\text{H}}_{2}{\text{O}}_{2,\text{T}}}{\text{C}}_{{\text{H}}_{2}{\text{O}}_{2},\text{T}}}{{\text{k}}_{\text{HO}\bullet \text{i}}{\text{C}}_{\text{i}}+{\text{k}}_{\text{HO}\bullet {\text{H}}_{2}{\text{O}}_{2,\text{T}}}{\text{C}}_{{\text{H}}_{2}{\text{O}}_{2,\text{T}}}}\right)\frac{{\text{I}}_{0}{\text{C}}_{\text{i}}\left[1-\text{exp}(-2.303\text{LA})\right]}{\text{A}}$$

**Fig. 4:**
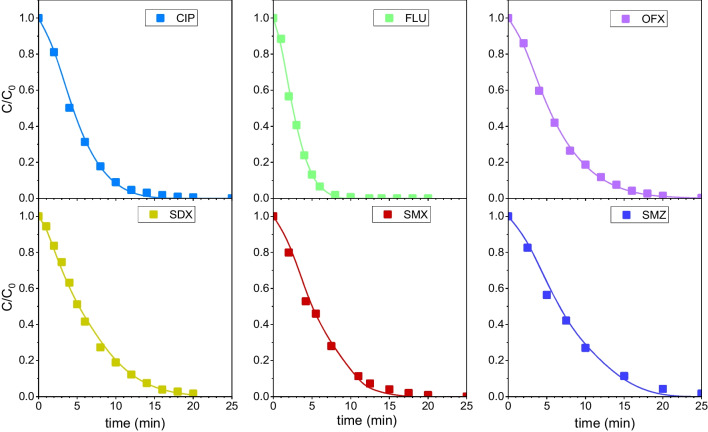
Evolution of the dimensionless concentration of fluoroquinolone and sulfonamide antibiotics with time. Determination of the hydroxyl radical rate constant according to method A. Symbols: Experimental data; Lines: fittings to Eq. (27); Conditions: C_0_ = 10^–4^ M, C_H2O2,0_ = 0.1 M, I_0_ = 6.9 × 10^–6^ E·L^−1^ s^−1^, L = 2.46 cm, pH 7 (H_3_PO_4_ = 0.05 M)

Batch UV_C_/H_2_O_2_ experiments were carried out with individual antibiotics measuring C_i_, C_H2O2,T_ and A at different sampling times. Then, data were fitted to Eq. (27) by a non-linear least squares regression to obtain k_HO•,i_. Figure 4 depicts graphical results of the regression analysis for some antibiotics while the computed k_HO•,i_ values are shown in Table 2 under Method A.

**Table 2:** k_HO•,i_ and k^1^_O₂,i_ values determined in this work

	Method A^a^	Method B^b^	Method C^c^	
Antibiotic	k_HO•,i_ × 10^–9^, M^−1^ s^−1^	k_HO•,i_ × 10^–9^, M^−1^ s^−1^	k_HO•,i_ × 10^–9^, M^−1^ s^−1^	k^1^_O₂,i_ × 10^–6^, M^−1^ s^−1^
AMP	n.d	10.6	9.54	9
CFX	n.d	21.8	17.0	8
CIP	2.47	12.4	10.4	7.5
FLU	4.01	20.0	15.1	1.5
MTZ	n.d	4.21	3.80	6
OFX	1.68	8.67	7.58	50
OXT	n.d	8.04	6.93	1
SDX	1.39	9.41	7.52	50
SMX	1.55	9.32	7.38	5
SMZ	1.21	6.98	6.07	9
TMP	1.34	6.46	5.49	5
TTC	n.d	8.34	8.57	50
TYL	n.d	10.6	8.20	30

n.d.: not determined; ^a^ Numerical method according to the Eq. (27); ^b^ Competitive method without accounting for the contribution of direct photolysis (28);^c^ Competitive method accounting for the contribution of direct photolysis, Eq. (30)

**Fig. 5:**
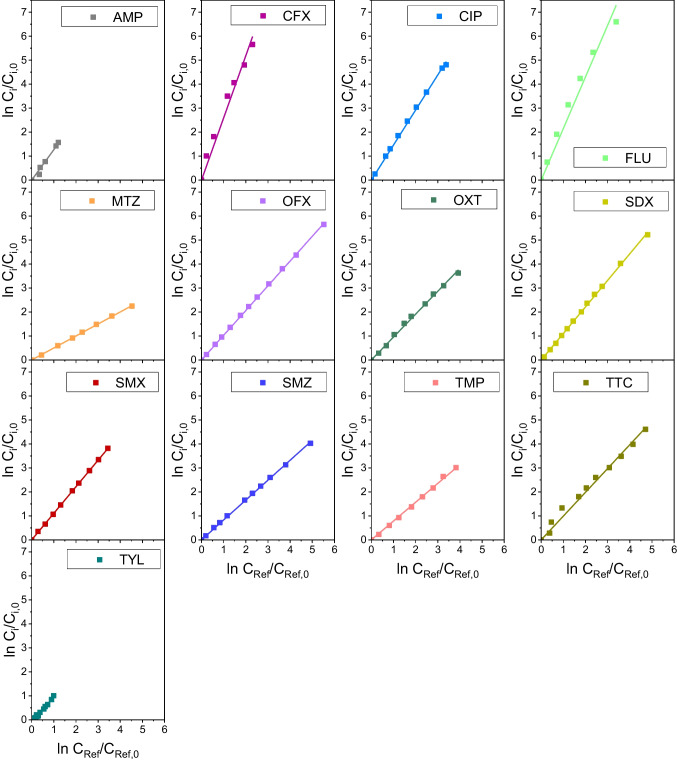
Determination of the individual hydroxyl radical rate constant according to method B. Reference compound: phenol. Symbols: Experimental concentration; Lines: linear fittings to Eq. (28). Conditions: C_i0_ = 10^–4^ M, C_Ref 0_ = 10^–4^ M, C_H2O2 0_ = 0.1 M, I_0_ = 6.9 × 10^–6^ E L^−1^ s^−1^, L = 2.46 cm, pH 7 (H_3_PO_4_ = 0.05 M)

Despite the fact that the method described above does not require the use of any reference compound, thus avoiding potential errors associated to reference compound data, it has received some criticism as usually underestimates k_HO•,i_ (Wojnárovits et al. 2018). The UV_C_/H_2_O_2_ competitive kinetics method is straightforward if the contribution of direct photolysis (i.e., r_UV_) is considered negligible in the mass balance Eq. (21), for both the target and the reference compounds. Then, Eq. (28) applies (Bahnmüller et al. 2015):

28 $$\text{ln}\frac{{\text{C}}_{\text{i}}}{{\text{C}}_{\text{i}0}}=\frac{{\text{k}}_{\text{HO}\bullet ,\text{i}}}{{\text{k}}_{\text{HO}\bullet ,\text{Ref}}}\text{ln}\frac{{\text{C}}_{\text{Ref}}}{{\text{C}}_{\text{Ref }0}}$$

where the subscript Ref denotes the reference compound. From Eq. (28) a plot of its left-hand side term against ln(C_Ref_/C_Ref 0_) should yield a straight line. Figure 5 shows such plots for data obtained with the antibiotics investigated using phenol as the reference compound. In most of the cases good linear behavior of experimental data was observed (R^2^ > 0.985). From the slopes of the best-fitting straight lines (i.e., least squares regression), k_HO•,i_ values were obtained (see Table 2, method B) given that k_HO•,Ref_ = 8.41 × 10^9^ M^−1^ s^−1^ was known for phenol (Wojnárovits et al. 2018).

Experimental data from UV_C_/H_2_O_2_ competitive kinetics runs were also used to compute k_HO•,i_ without neglecting the contribution of direct photolysis in Eq. (21). First, the actual concentration of hydroxyl radical at any time was obtained after applying Eq. (21) to phenol:

29 $${\mathrm{C}}_{\mathrm{HO}\bullet }=\frac{-\frac{{\mathrm{dC}}_{\mathrm{Ref}}}{\mathrm{dt}}-\frac{{\mathrm{I}}_{0}{\Phi }_{\mathrm{Ref}}{\upvarepsilon }_{\mathrm{Ref}}{\mathrm{C}}_{\mathrm{Ref}}}{\mathrm{A}}\left[1-\mathrm{exp}(-2.303\mathrm{LA})\right]}{{\mathrm{k}}_{\mathrm{HO}\bullet ,\mathrm{Ref}}{\mathrm{C}}_{\mathrm{Ref}}}$$

Then, Eq. (29) was substituted into the mass balance equation of antibiotic *i* to yield:

30 $$-\frac{{\mathrm{dC}}_{\mathrm{i}}}{\mathrm{dt}}=\frac{{\mathrm{I}}_{0}{\Phi }_{\mathrm{i}}{\varepsilon }_{\mathrm{i}}{\mathrm{C}}_{\mathrm{i}}}{\mathrm{A}}\left[1-\mathrm{exp}\left(-2.303\mathrm{LA}\right)\right]+{\mathrm{k}}_{\mathrm{HO}\bullet ,\mathrm{i}}{\mathrm{C}}_{\mathrm{i}}\frac{-\frac{{\mathrm{dC}}_{\mathrm{Ref}}}{\mathrm{dt}}-\frac{{\mathrm{I}}_{0}{\Phi }_{\mathrm{Ref}}{\varepsilon }_{\mathrm{Ref}}{\mathrm{C}}_{\mathrm{Ref}}}{\mathrm{A}}\left[1-\mathrm{exp}(-2.303\mathrm{LA})\right]}{{\mathrm{k}}_{\mathrm{HO}\bullet ,\mathrm{Ref}}{\mathrm{C}}_{\mathrm{Ref}}}$$

**Fig. 6:**
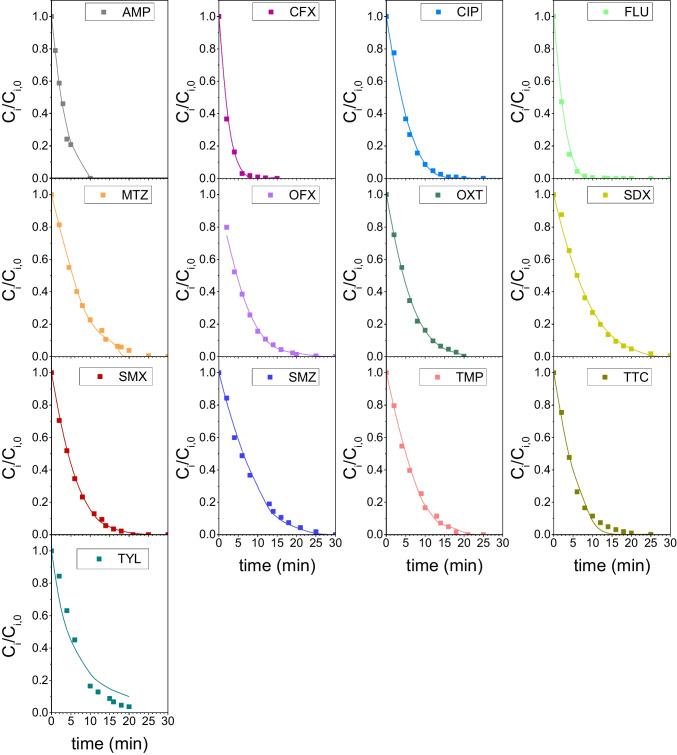
Evolution of the dimensionless concentration of antibiotics with time during individual UV_C_/H_2_O_2_ experiments. Determination of the individual hydroxyl radical rate constant by the competitive method according to method C. Symbols: Experimental concentration; Lines: fittings to Eq. (29). Conditions as in Fig. 5

Removal rates of the antibiotic (dC_i_/dt) and phenol (dC_Ref_/dt) were estimated by the finite difference method applied to the experimental concentration–time data (i.e., UV_C_/H_2_O_2_ run) while the molar absorption coefficient and the direct quantum yield of phenol at pH 7 were obtained from separated experiments (ε_Ref=_ 0.4 mM^−1^ cm^−1^; Φ_Ref_ = 2.2 mmol·E^−1^, see Fig. S1). The values shown in Table 1 for the molar absorption coefficient (ε) and the direct quantum yield (Φ_d_) of antibiotics were used for ε_i_ and Φ_i_ in Eq. (30). Then, experimental data were fitted to the Eq. (30) by a non-linear regression method to minimize MSE (Fig. 6). The only unknown in the Eq. (30), k_HO•,i_ was thus obtained (see Table 2, method C). Typically, deviations lower than 20% between calculated k_HO•,i_ (method C) and the average of those reported in the literature (see Table S1) were found for the antibiotics, though larger differences were found for FLU and OFX.

As seen in Table 2, significant differences were observed between the values of k_HO•,i_ computed by the three methods used. First, values obtained after applying the direct method (method A) were noticeably lower than those derived from the competitive methods. This fact has also been observed by other researchers (Wojnárovits et al. 2018). Regarding the competitive method, neglecting the contribution of the direct photolysis to the overall antibiotic removal led to overestimated values of k_HO•,i_. Consequently, k_HO•,i_ values determined by the method C were considered as more suitable for further kinetic modelling purposes.

### Kinetic modelling

A second part of this work focusses on the kinetic modelling of the removal of an aqueous mixture of the thirteen antibiotics by UV_C_ and UV_C_/H_2_O_2_ processes. For that purpose, a set of ODEs (Eqs. (10) and (11)) was adopted to describe the temporal dynamics of each antibiotic and hydrogen peroxide in a completely mixed batch photoreactor. Lamp and photoreactor characteristics (i.e., I_0_ and L), molar absorption coefficients, direct quantum yields and hydroxyl radical rate constants were taken as determined above (see Experimental section and Table 1). Also, the rate constants of the singlet oxygen reactions, k^1^_O₂_, were determined as shown below. The contribution of TPs to the overall absorbance at 254 nm of reaction samples measured with 1 cm quartz cell was experimentally demonstrated negligible, as the condition (31) fulfilled:

31 $$\mathrm{A}\approx \sum_{\mathrm{i}}{\varepsilon}_{\mathrm{i}}{\mathrm{C}}_{\mathrm{i}}$$

where *i* stands for any parent antibiotic and hydrogen peroxide (if added) but not for reaction products. Therefore, Σε_i_C_i_ was used in Eq. (10) instead of experimental values of A for computing purposes.

In the absence of added H_2_O_2_ (i.e., UV_C_ modelling) the concentration of HO^•^ was assumed to be low enough to make negligible the third term of the right-hand side of Eq. (10). However, given the presence of photosensitizing antibiotics (i.e., fluoroquinolones), singlet oxygen might have a major impact on antibiotics degradation. The concentration of singlet oxygen was obtained at any reaction time of UV_C_ runs from Eq. (32), where OFX is chosen as the reference compound since the value of the rate constant of the reaction between OFX and singlet oxygen is known to be k^1^_O₂,OFX_ = 5.6 × 10^6^ M^−1^ s^−1^ (Albini and Monti 2003):

32 $${\mathrm{C}}_{{1}_{{0}_{2}}}=-\frac{\frac{{\mathrm{dC}}_{\mathrm{OFX}}}{\mathrm{dt}}+\frac{{\mathrm{I}}_{0}{\Phi }_{\mathrm{OFX}}{\varepsilon }_{\mathrm{OFX}}{\mathrm{C}}_{\mathrm{OFX}}}{\sum_{\mathrm{i}}{\varepsilon }_{\mathrm{i}}{\mathrm{C}}_{\mathrm{i}}}\left[1-\mathrm{exp}(-2.303\mathrm{L}\sum_{\mathrm{i}}{\varepsilon }_{\mathrm{i}}{\mathrm{C}}_{\mathrm{i}})\right]}{{\mathrm{k}}_{{1}_{{0}_{2}},\mathrm{OFX}}{\mathrm{C}}_{\mathrm{OFX}}}$$

For the UV_C_/H_2_O_2_ system, however, the HO^•^ oxidation pathway prevails because of the substantial formation of HO^•^ by photolytic cleavage of H_2_O_2_. Accordingly, the second term of the right-hand side of Eq. (10) (i.e., contribution of singlet oxygen reactions to antibiotic removal) can be considered as negligible. Then, one of the antibiotics in the mixture was used as a reference compound (Ref) to estimate the actual concentration of hydroxyl radical at any time of UV_C_/H_2_O_2_ runs:

33 $${\mathrm{C}}_{{\mathrm{HO}}^{\bullet }}=-\frac{\frac{{\mathrm{dC}}_{\mathrm{Ref}}}{\mathrm{dt}}+\frac{{\mathrm{I}}_{0}{\Phi }_{\mathrm{Ref}}{\varepsilon }_{\mathrm{Ref}}{\mathrm{C}}_{\mathrm{Ref}}}{\sum_{\mathrm{i}}{\varepsilon }_{\mathrm{i}}{\mathrm{C}}_{\mathrm{i}}}\left[1-\mathrm{exp}(-2.303\mathrm{L}\sum_{\mathrm{i}}{\varepsilon }_{\mathrm{i}}{\mathrm{C}}_{\mathrm{i}})\right]}{{\mathrm{k}}_{{\mathrm{HO}}^{\bullet },\mathrm{Ref}}{\mathrm{C}}_{\mathrm{Ref}}}$$

**Fig. 7:**
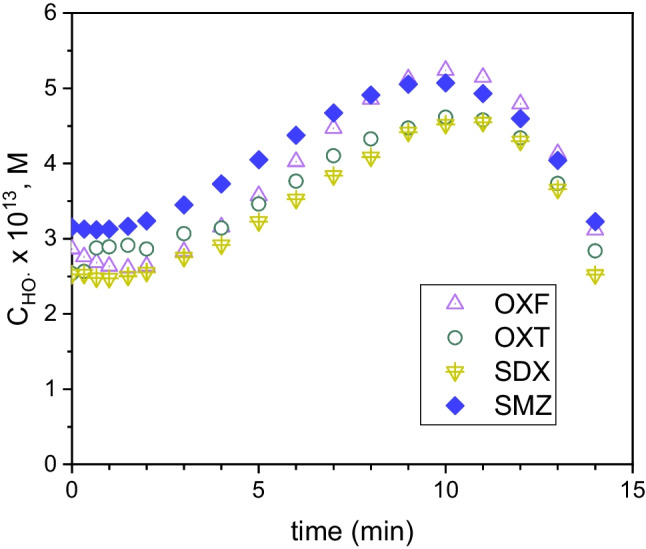
Variation with time of the calculated concentration of HO^•^ using different antibiotics as the reference compound in Eq. (33)

It is important to recall that Eqs. (32) and (33) estimate the actual concentration of ROS as they are based on experimental kinetic data of a reference compound. In this sense, the ROS scavenging effects of TPs and background water matrix is inherently considered. As an example, Fig. 7 shows the time evolution of the concentration of hydroxyl radical during a fourteen-minute UV/H_2_O_2_ run as computed by Eq. (33) using different antibiotics as Ref. It can be seen that similar profiles were obtained regardless of the species used as the reference compound.

A Matlab (MathWorks) code was written to solve the set of ordinary differential Eqs. (29) (one for each antibiotic) applying the Ode45 solver. In the case of the UV_C_ kinetic model, a regression algorithm was used to find k^1^_O₂-i_ values that minimize the MSE between experimental and calculated antibiotic concentration profiles. Table 2 shows the final rate constants achieved by this regression method, which were in the 1–50 μM^−1^ s^−1^ range as typically found in the literature for most of these antibiotics (Albini and Monti 2003; Boreen et al. 2005; Luo et al. 2012; Li et al. 2016; Lian et al. 2017; Ge et al. 2019; Tang et al. 2023). Also, it can be seen in Table 2 that k_HO•,i_ values are much larger than the corresponding k^1^_O₂,i_, indicating higher reactivity of HO^•^ than ^1^O_2_ with any of the antibiotics studied in this work. However, the concentration of ^1^O_2_ in water is expected to be some orders of magnitude higher than HO^•^ upon UV_C_ irradiation (Vione et al. 2010), likely making the ^1^O_2_-mediated degradation of antibiotics the primary indirect photo-oxidation mechanism.

**Fig. 8:**
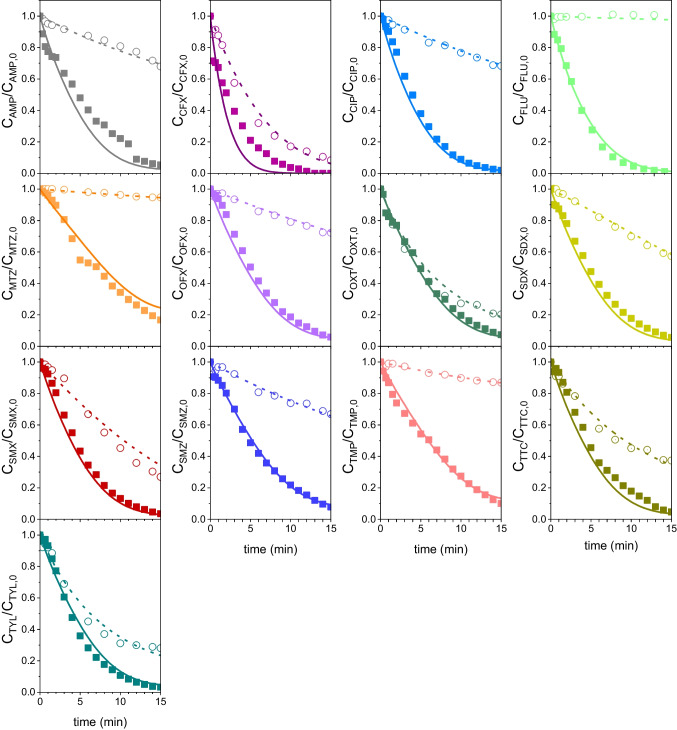
Evolution of dimensionless concentration of each antibiotic with time during UV_C_ irradiation runs (dotted curves and open circles) and UV_C_/H_2_O_2_ oxidation runs (solid curves and solid squares) treating a mixture of the thirteen antibiotics in water. SMZ was used as reference compound to compute C_HO•_ by Eq. (33) Symbols: experimental concentrations. Curves: calculated concentrations from kinetic models using data from Table 1 and Table 2 as input parameters. Other conditions: C_i0_ = 10^–5^ M each antibiotic, C_H2O2 0_ = 0.01 M (when needed), I_0_ = 6.9 × 10^–6^ E·L^−1^ s^−1^, L = 2.46 cm, pH 7 (H_3_PO_4_ = 0.05 M)

Figure 8 compares the experimental and simulated concentration–time profiles of each antibiotic in the aqueous mixture during UV_C_ and UV_C_/H_2_O_2_ runs. The C_HO•_ values were computed by Eq. (33) using SMZ as the reference compound. Nevertheless, similar modelling results were obtained using other antibiotics as the reference compound (see Fig. S2 for CIP and OFX profiles as examples). The good concordance between experimental and simulated UV_C_ data supports the values of the direct quantum yields obtained in this work (Table 1) and the fact that the contribution of singlet oxygen reactions to the overall antibiotic removal cannot be neglected in the kinetic modelling of the UV_C_ oxidation system. Figure 8 also shows that the computed values for the UV_C_/H_2_O_2_ system are in fairly good agreement with experimental data. This supports the values of k_HO•,i_ used for simulation purposes (Table 1, Method C) and the hypothesis considered in the UV_C_/H_2_O_2_ kinetic model (i.e., negligible contribution of singlet oxygen reactions to the antibiotic removal rate). As long as the UV/H_2_O_2_ AOP progressed, the antibiotics were degraded mainly by the oxidizing action of HO^•^, photogenerated from H_2_O_2_ decomposition. Therefore, aqueous H_2_O_2_ concentration decreased with the irradiation time. It should also be highlighted that the UV_C_/H_2_O_2_ kinetic model also led to good agreement between calculated and experimental concentrations of H_2_O_2_ with deviations below 5%.

## Conclusions

The removal of antibiotics in aqueous solution by UV_C_ and UV_C_/H_2_O_2_ processes in a completely mixed batch photoreactor can be successfully predicted by a simple, semi-empirical model, which accounts for direct photolysis and reactions of antibiotics with photogenerated ROS (i.e. hydroxyl radical and singlet oxygen). ROS scavenging and UV shielding effects of background water matrix and degradation products is inherently considered in the model by estimating the actual concentrations of ROS and the absorbance of the aqueous solution from experimental data. Reliable photochemical data such as photoreactor parameters (i.e., intensity of incident radiation and effective light path length), photochemical properties of the antibiotics and hydrogen peroxide (i.e., molar absorption coefficient and direct quantum yields) and the chemical reaction rate constants of the antibiotics with ROS species (i.e., hydroxyl radical and singlet oxygen) are crucial for a good model prediction. For the antibiotics used in this study, direct quantum yields varied from less than 0.01 mmol·E^−1^ (FLU and TMP) to about 70 mmol·E^−1^ (AMP), k_HO•,i_ values ranged from 3.8 × 10^9^ M^−1^ s^−1^ (MTZ) to 1.7 × 10^10^ M^−1^ s^−1^ (CFX) and k^1^_O₂,i_ values fell within the 10^6^ M^−1^ s^−1^ and 5 × 10^7^ M^−1^ s^−1^ limits.

## Supplementary Information

Below is the link to the electronic supplementary material.

Supplementary file1 (DOCX 95 KB)

## Data Availability

Data will be made available on reasonable request.
