# Supplementary material for: Kinetic modelling of UVC and UVC/H2O2 oxidation of an aqueous mixture of antibiotics in a completely mixed batch photoreactor
Source: Environ Sci Pollut Res Int. 2024 Sep 3;31(43):55222–38. doi: 10.1007/s11356-024-34812-7 (PMC11415419; doi:10.1007/s11356-024-34812-7)
Supplement: Supplementary file 1 — Supplementary file1 (DOCX 95 KB) [file 11356_2024_34812_MOESM1_ESM.docx]

**Supplementary information**

**Kinetic modelling of UV_C_ and UV_C_/H_2_O_2_ oxidation of an aqueous mixture of antibiotics in a completely mixed batch photoreactor**

Fernando J. Beltrán, Ana M. Chávez, Miguel A. Jiménez-López, Pedro M. Álvarez^*^

Departamento de Ingeniería Química y Química Física, Instituto Universitario del Agua, Cambio Climático y Sostenibilidad (IACYS), Universidad de Extremadura, 06006 Badajoz, Spain

^*^ Corresponding author: pmalvare@unex.es

**1. Literature data**

| Table S1. Literature data on some antibiotics at circumneutral pH: molar absorption coefficient (ε), quantum yield at 254nm (φ), rate constant of the reaction with singlet oxygen (k_¹O₂_) and rate constant of the reaction with hydroxyl radical (k_HO•_) | | | | |
| --- | --- | --- | --- | --- |
| **Antibiotic** | **ε**  **(mM^-1^·cm^-1^)** | Φ  **(mmol·E^-1^)** | **k_¹O₂_ × 10^-6^**  **(M^-1^s^-1^)** | **k_HO•_×10^-9^**  **(M^-1^s^-1^)** |
| Ampicillin (AMP) | 0.30 [1] | n.r. | n.r. | 4.87 [1]  8.2 [2] |
| Cefuroxime (CFX) | n.r. | n.r. | n.r. | 15 [3] |
| Ciprofloxacin (CIP) | 16.5 [4]  22.0^a^[5] | 10.3 [6] | 3.2 [7] | 4.1 [8]  21.5 [2] |
| Flumequine (FLU) | n.r. | n.r. | 3.4 [7] | 8.3 [8]  5.7 [10] |
| Metronidazole (MTZ) | 2.1 [5] | 3.5 [5] | 340 [11] | 4.1 [5]  3.54 [2] |
| Ofloxacin (OFX) | 11.9 [12] | 1.8 [12] | 5.6 [7] | 4.2 [12] |
| Oxytetracycline (OXT) | 11.6 [13]  14.3^a^ [5] | 9.8 [13]  4.2^a^ [5] | 128 [14] | 7.18 [13]  5.6 [2] |
| Sulfadimethoxine (SDX) | 16.9^b^ [15] | 36.0 [15] | 190^a^ [16] | 6.46^a^ [2] |
| Sulfamethoxazole (SMX) | 16.8 [5] | 46.0 [5] | 0.02 [17] | 5.5 [5]  6.50^a^ [2] |
| Sulfamethazine (SMZ) | 18.5^c^ [5] | 8.7^c^ [5] | 150 [18]  1720^a^ [16] | 5.65^c^ [5]  8.81 [2] |
| Trimethoprim (TMP) | 2.94^c^ [5] | 1.2^c^ [5] | 3.2 [19] | 6.9 [8] |
| Tetracycline (TTC) | 13.5^a^ [5] | 3.8^a^ [5] | n.r. | 7.7 [8] |
| Tylosin (TYL) | 3.87 [20] | 30-40 [20] | n.r. | 8.2 [8] |

Data at pH 7 unless otherwise indicated: ^a^ pH=8; ^b^ pH=6.2; ^c^ pH=7.8; n.r.: not reported.

[1] He X, Mezyk SP, Michael I, Fatta-Kassinos D, Dionysiou DD (2014) Degradation kinetics and mechanism of β-lactam antibiotics by the activation of H_2_O_2_ and Na_2_S_2_O_8_ under UV-254 nm irradiation, J Hazard Mater. <https://doi.org/10.1016/J.JHAZMAT.2014.07.008>

[2] Wojnárovits L, Tóth T, Takács E (2018) Critical evaluation of rate coefficients for hydroxyl radical reactions with antibiotics: A review. Crit Rev Environ Sci Technol. <https://doi.org/10.1080/10643389.2018.1463066>

[3] Mandal S, (2018) Reaction rate constants of hydroxyl radicals with micropollutants and their significance in advanced oxidation processes J Adv Oxid Technol. <https://doi.org/10.26802/jaots.2017.0075>

[4] Snowberger S, Adejumo H, He K, Mangalgiri KP, Hopanna M, Soares AD, Blaney L (2016). Direct Photolysis of fluoroquinolone antibiotics at 253.7 nm: specific reaction kinetics and formation of equally potent fluoroquinolone antibiotics. Env. Sci. Technol. <https://doi.org/10.1021/acs.est.6b01794>

[5] Wols BA, Hofman-Caris CHM, Harmsen DJH, Beerendonk EF (2013) Degradation of 40 selected pharmaceuticals by UV/H_2_O_2_. Water Res.

<https://doi.org/10.1016/J.WATRES.2013.07.008>

[6] Pereira VJ, Weinberg HS, Linden KG, Singer PC (2007) UV degradation kinetics and modeling of pharmaceutical compounds in laboratory grade and surface water via direct and indirect photolysis at 254 nm. Environ Sci Technol. <https://doi.org/10.1021/es061491b>

[7] Albini A, Monti S (2003) Photophysics and photochemistry of fluoroquinolones. Chem Rev <https://doi.org/10.1039/B209220B>

[8] Dodd MC, Buffle MO, Von Gunten U (2006) Oxidation of antibacterial molecules by aqueous ozone: moiety-specific reaction kinetics and application to ozone-based wastewater treatment, Environ Sci Technol. <https://doi.org/10.1021/es051369x>

[9] Santoke H, Song W, Cooper WJ, Greaves J, Miller GE (2009) Free-Radical-Induced Oxidative and Reductive Degradation of Fluoroquinolone Pharmaceuticals: Kinetic Studies and Degradation Mechanism. J Phys Chem A. <https://doi.org/10.1021/jp9029453>

[10] Iqbal J et al (2020) Deep eutectic solvent-mediated synthesis of ceria nanoparticles with the enhanced yield for photocatalytic degradation of flumequine under UV-C. J Water Process Eng. <https://doi.org/10.1016/J.JWPE.2019.101012>

[11] Lian L, Yao B, Hou S, Fang J, Yan S, Song W (2017) Kinetic Study of Hydroxyl and Sulfate Radical-Mediated Oxidation of Pharmaceuticals in Wastewater Effluents. Environ Sci Technol.

<https://doi.org/10.1021/acs.est.6b05536>

[12] Márquez G, Rodríguez EM, Beltrán FJ, Álvarez PM (2013) Determination of rate constants for ozonation of ofloxacin in aqueous solution. Ozone Sci Eng. <https://doi.org/10.1080/01919512.2013.771530>

[13] Liu Y, He X, Duan X, Fu Y, Dionysiou DD (2015) Photochemical degradation of oxytetracycline: Influence of pH and role of carbonate radical. Chem Eng J. <https://doi.org/10.1016/J.CEJ.2015.04.048>

[14] Tang L, Zhou S, Li F, Sun L, Lu H (2023) Ozone micronano-bubble-enhanced selective degradation of oxytetracycline from production wastewater: The overlooked singlet oxygen oxidation. Environ Sci Technol. <https://doi.org/10.1021/acs.est.2c06008>

[15] Boreen AL, Arnold WA, McNeill K (2005) Triplet-sensitized photodegradation of sulfa drugs containing six-membered heterocyclic groups: Identification of an SO_2_ extrusion photoproduct. Environ Sci Technol. <https://doi.org/10.1021/es048331p>

[16] Ge L, Zhang P, Halsall C, Li Y, Chen CE, Li J, Sun H, Yao Z (2019) The importance of reactive oxygen species on the aqueous phototransformation of sulfonamide antibiotics: kinetics, pathways, and comparisons with direct photolysis. Water Res. <https://doi.org/10.1016/j.watres.2018.11.009>

[17] Boreen AL, Arnold WA, McNeill K (2004) Photochemical fate of sulfa drugs in then aquatic environment: Sulfa drugs containing five-membered heterocyclic groups. Environ Sci Technol. <https://doi.org/10.1021/es0353053>

[18] Li J, Zhang P, Ju MW, Yu CY, Ge LK, Na GS, Yao Z (2016) Kinetics and pathways for aqueous ROS photooxidation of sulfamethazine. Chin Sci Bull. <https://doi.org/10.1360/N972016-00140>

[19] Luo X, Zheng Z, Greaves J, Cooper WJ, Song W (2012) Trimethoprim: Kinetic and mechanistic considerations in photochemical environmental fate and AOP treatment. Water Res. <https://doi:10.1016/j.watres.2011.12.052>

[20] Voigt M, Jaeger M (2017) On the photodegradation of azithromycin, erythromycin and tylosin and their transformation products – A kinetic study, Sustain Chem Pharm. <https://doi.org/10.1016/j.scp.2016.12.001>

**2. Determination of apparent and direct quantum yields of phenol**

**Figure S1.** (A) Plot to determine quantum yields of phenol according to equation (20). (B) Normalized concentration of phenol during batch UV runs. Symbols: experimental data; Lines: calculated data using quantum yields as determined from plot A. Experimental conditions of batch UV runs: T = 18-20 ºC, pH 7, C_0_ = 0.1 mM, I_0_ = 6.9×10^-6^ Einstein·L^-1^·s^-1^, L = 2.46 cm. Open symbols: absence of ROS scavengers; Solid symbols: presence of sodium azide (0.01 M)

Equation (20):

(20)

**3. Validation of the UV_C_/H_2_O_2_ kinetic model using different reference compounds to determine the concentration of HO^•^**

**Figure S2**. Evolution of dimensionless concentration of CIP and OFX with time during UV_C_/H_2_O_2_ oxidation runs treating a mixture of the thirteen antibiotics in water. Different antibiotics were used as the reference compound in equation (31) to determine actual concentration of hydroxyl radical. Symbols: Experimental data. Curves: calculated data from the kinetic model. CIP (blue squares and straight lines) and OFX (purple triangles and dashed lines). Other conditions: C_0_ =10^-5^ M each antibiotic, C_H2O2,0_ = 0.01 M, I_0_= 6.9×10^-6^ E·L^-1^s^-1^, L = 2.46 cm, pH 7 (H_3_PO_4_= 0.05 M)
